# Higher circulating FGF21, lower protein intake, and lower muscle mass: Associations with a higher risk of mortality

**DOI:** 10.1111/joim.20099

**Published:** 2025-05-19

**Authors:** Adrian Post, Wendy A. Dam, Dion Groothof, Casper F. M. Franssen, Stephan J. L. Bakker, Robin P. F. Dullaart

**Affiliations:** ^1^ Internal Medicine Department University Medical Center Groningen University of Groningen Groningen The Netherlands

**Keywords:** all‐cause mortality, fibroblast growth factor 21, general population, muscle mass, protein intake

## Abstract

**Background and Objectives:**

This population‐based study explores associations of fibroblast growth factor 21 (FGF21), a key modulator of processes linked to protein metabolism, with protein intake and muscle mass, and their relationships with all‐cause mortality.

**Methods:**

In 6395 participants (mean age 54 years; 50% women), circulating FGF21 (immunoassay), protein intake (Maroni equation using 24‐h urinary urea excretion; low intake defined as <0.8 g/kg/day), and muscle mass (24‐h creatinine excretion rate indexed to height squared (CERI)) were documented.

**Results:**

FGF21 concentration was 896 (540–1384) pg/mL, protein intake was 1.01 (0.85–1.19) g/kg/day, and CERI was 4.1 ± 0.9 mmol/day/m^2^. Higher FGF21 was associated with higher odds of low protein intake (odds ratio per doubling: 1.48; 95% confidence interval [CI]: 1.38–1.58; *p* < 0.0001) and lower muscle mass (standardized beta: −0.08; 95% CI: −0.10; −0.06; *p* < 0.001). Over 10.4 years of follow‐up, 955 deaths were registered. Higher FGF21 was associated with increased mortality (hazard ratio (HR) per doubling: 1.09; 95% CI: 1.02–1.16; *p* = 0.009). Conversely, higher protein intake (HR per doubling: 0.67; 95% CI: 0.56–0.81; *p* < 0.0001) and higher CERI (HR per standard deviation increase: 0.83; 95% CI: 0.76–0.90; *p* < 0.0001) were associated with a lower risk of mortality, independent of potential confounders. However, the FGF21‐mortality association became non‐significant after adjusting for protein intake.

**Conclusion:**

Higher FGF21 was associated with higher odds of low protein intake. The observed association of higher FGF21 concentrations and risk mortality was predominantly attributable to lower protein intake. In contrast, both higher protein intake and higher muscle mass were independently associated with lower mortality risk, highlighting the potential relevance of protein intake and maintenance of muscle mass in long‐term health.

## Graphical abstract

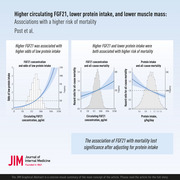

## Introduction

Fibroblast growth factor 21 (FGF21) plays a crucial role in metabolic regulation and is involved in energy homeostasis, nutrient metabolism, and muscle function. Its increase in reaction to nutritional stress, such as protein restriction or fasting, positions FGF21 as a central player in the body's adaptive response to nutrient scarcity [1]. Both animal and human studies have shown that protein restriction significantly elevates circulating FGF21 concentrations, independent of energy intake, indicating its role in maintaining metabolic balance during periods of reduced protein availability [1, 2].

In addition to its role in nutrient metabolism, FGF21 has been implicated in muscle atrophy. Under protein‐restricted conditions, FGF21 appears to play a dual role: on the one hand, it promotes amino acid homeostasis by conserving nutrients and optimizing their availability; on the other hand, chronic FGF21 exposure can trigger muscle catabolism through decreased protein synthesis and increased protein degradation. This seemingly paradoxical effect—preserving essential amino acids while facilitating protein breakdown—likely reflects an adaptive mechanism to ensure survival during prolonged protein scarcity [3].

Elevated FGF21 concentrations are commonly observed in metabolic disorders, including diabetes, insulin resistance, and obesity, and have been associated with increased mortality in conditions like coronary disease, chronic kidney disease, cancer, and sepsis [4, 5, 6]. Recent evidence suggests a link between higher FGF21 concentrations and a higher risk of mortality in the general population, although the underlying mechanisms are not fully understood. [7]. Nutritional status, including protein intake and muscle mass, may play key roles in this relationship. Low muscle mass, which may partly mediate the association between protein intake and mortality, is consistently recognized as a strong predictor of increased mortality risk [8]. However, the direct relationship between protein intake and mortality remains unclear, with studies reporting conflicting results [9, 10]. The interplay between FGF21, protein intake, and muscle mass, and their combined influence on mortality, has not been thoroughly investigated in humans. Understanding these interrelations could provide insights into potential mechanisms underlying the association between higher FGF21 concentrations and long‐term health outcomes.

In this population cohort study, the objective was to investigate (1) the association of circulating FGF21 concentration with the odds of low protein intake, (2) the continuous associations of FGF21 concentration with protein intake and muscle mass, and (3) the associations of FGF21, protein intake, and muscle mass with all‐cause mortality, while also exploring the interrelations in their association with mortality.

## Methods

### Study design and participants

This study was conducted as part of the Prevention of Renal and Vascular End‐stage Disease (PREVEND) study, a prospective observational cohort designed to assess the prevalence and implications of microalbuminuria in adults living in Groningen, the Netherlands. Detailed descriptions of the study's design and objectives have been previously published [11]. In short, inhabitants of Groningen between 28 and 75 years old (*n* = 85,421) received an invitation to participate in 1997–1998. Residents were asked to provide a first‐morning urine sample and fill in a short questionnaire, to which 47.8% of the individuals responded. Following further exclusions based on willingness or eligibility to participate, the final PREVEND cohort consisted of 8592 participants. A follow‐up screening phase occurred between 2001 and 2003, involving 6894 participants, which served as the starting point for the present analysis. From this group, 499 individuals were excluded due to missing data on circulating FGF21 concentrations, protein intake, or muscle mass, resulting in a final study population of 6395 participants. Missing values for covariates were addressed using imputation, as detailed in the methods section. A visual representation of participant selection and inclusion is available in Fig. S1. The PREVEND study received ethical approval (MEC 96/01/022) and was conducted following the principles outlined in the Declaration of Helsinki. All individuals provided written informed consent prior to participation, and the study adheres to the STROBE guidelines.

### Assessments

Blood pressure readings were obtained from the right arm using an automatic device (Dinamap XL Model 9300). To collect baseline biochemical data, EDTA plasma samples were drawn from all participants between 8:00 and 10:00 a.m. These samples were directly aliquoted and stored at −80°C for future analysis. Participants were required to collect two consecutive 24‐h urine samples after receiving detailed instructions. Once collected, urine samples were kept refrigerated at 4°C for up to 4 days before the second study visit. Afterwards, aliquots were stored at −20°C until laboratory analysis. CirculFGF21 concentrations were quantified using an immunoassay as previously described [12].

Protein intake was assessed using 24‐h urea excretion and the Maroni equation [13]:

Protein intake (g/kg/day) = (6.25 × (0.028 × urinary urea excretion + 0.031 × body weight) + urinary protein excretion)/bodyweight. A protein intake <0.8 g/kg/day was considered a low protein intake. Sensitivity analyses were included using alternative cut‐offs of <0.6 and <1.0 g/kg/day.

Muscle mass was estimated using the creatinine excretion rate index (CERI) [14], calculated as follows:

CERI (mmol/24‐h/m^2^) = CER/height^2^, in which CER is the urinary creatinine excretion, averaged from two 24‐hour urinary collections. As no validated cut‐offs are established yet to define low muscle mass with CERI, we only performed analyses with muscle mass using CERI as a continuous variable. The glomerular filtration rate was estimated using the CKD‐EPI equation from 2021 using both creatinine and cystatin C [15]. Lipids, triglycerides, glucose, insulin, albumin, C‐reactive protein, aminotransferases, gamma‐GT, creatinine, and cystatin C were measured using standard protocols, as previously documented for this cohort [16, 17, 18, 19]. In sensitivity analyses, the fatty liver index (FLI) and hepatic steatosis index were utilized as surrogate markers for metabolic dysfunction‐associated steatotic liver disease (MASLD) [20]. The FLI was determined using the equation:

FLI = 100 × (e^(0.953×ln(triglycerides)+0.139×BMI+0.718×ln(GGT)+0.053× waist−15.745))/(1 + e^(0.953×ln(triglycerides)+0.139×BMI+0.718×ln(GGT)+0.053×waist−15.745)). Hepatic steatosis index (HSI) was calculated using the equation:

HSI = 8 × ALAT/ASAT + BMI + 2 (in case of comorbid type 2 diabetes) + 2 (if female).

Ketone levels and glycoprotein acetylation (GlycA) were assessed using a fully automated, high‐throughput 400 MHz proton (^1H) nuclear magnetic resonance (NMR) spectroscopy platform [21, 22]. GlycA represents a composite NMR signal that reflects both the concentration and glycosylation patterns of acute‐phase proteins, reflecting as an indicator of broader inflammatory activity. Homeostatic model assessment for insulin resistance (HOMA‐IR) was calculated as the product of insulin and fasting plasma glucose divided by 22.5 and used as a marker for insulin resistance [23]. A history of cardiovascular disease was defined as having a history of coronary artery disease, stroke, heart failure, and/or peripheral artery disease. Type 2 diabetes was classified according to the criteria established by the American Diabetes Association [24].

### Survival data

Information on all‐cause mortality was obtained from Statistics Netherlands. Follow‐up duration was calculated from the date of the second screening round (baseline) until the occurrence of an event, defined as death, loss to follow‐up, or the study's endpoint (January 1, 2017), whichever came first. For individuals who relocated to an unknown address, the date they were removed from the municipal registry was recorded as the censoring date. The cause of death was ascertained by linking the death certificate number to the underlying cause of death, as coded by a physician at Statistics Netherlands (CBS), in accordance with the 10th revision of the International Classification of Diseases. The primary analyses were conducted using all‐cause mortality. Sensitivity analyses were performed for cardiovascular mortality and non‐cardiovascular mortality.

### Statistical analyses

All statistical analyses were conducted using R version 4.3.0. Depending on the data distribution, continuous variables were reported as mean ± standard deviation (SD) for normally distributed data, median (Q1–Q3) for skewed distributions, and categorical variables as counts with percentages. To assess the associations of age with FGF21 concentrations, protein intake, and CERI, we compared distributions between participants aged <65 and ≥65 years using both the Wilcoxon rank‐sum test and the independent samples *t*‐test, depending on the normality of the data. In addition, Pearson correlation analyses were performed to evaluate continuous associations with age.

To minimize potential bias from missing data [25], multiple imputations were performed using the “mice” package, generating five imputed datasets. Statistical analyses were conducted separately within each imputed dataset, and pooled estimates were generated following Rubin's rules [25]. To examine the relationship between circulating FGF21 levels and low protein intake (<0.8 g/kg/day), logistic regression analyses were performed. Due to the skewed distribution, FGF21 was log_2_‐transformed prior to analyses. Linearity was assessed by comparing the fit of linear and non‐linear models. Logistic regression models were adjusted for predefined confounders, including age, sex, estimated glomerular filtration rate (eGFR), urinary albumin excretion, body mass index, smoking status, alcohol intake, diabetes, hypertension, history of cardiovascular disease, lipids, and plasma albumin. Additionally, stratified analyses were performed according to age groups <65 years or ≥65 years. To test the robustness of associations, sensitivity analyses were performed in which the following groups were excluded: (1) participants with FGF21 levels in the highest or lowest 2.5 percentiles, (2) those with type 2 diabetes, (3) individuals with body mass index <18.5 or >30 kg/m^2^, (4) those with eGFR < 60 mL/min/1.73 m^2^, and (5) participants with a history of cardiovascular disease. As FGF21 has been linked to muscle mass, MASLD, ketone metabolism, insulin resistance, and inflammation, additional models were adjusted for CERI, ketone body concentrations, FLI, HSI, HOMA‐IR, high‐sensitivity C‐reactive protein (hs‐CRP), and GlycA. Furthermore, we performed additional adjustments for potential confounders, including asthma or chronic obstructive pulmonary disease (COPD), rheumatic disease, and a history of malignancy. Lastly, we included sensitivity analyses using alternative low protein intake cut‐offs of 0.6 and 1.0 g/kg/day.

Linear regression models were used to explore associations between FGF21 and both protein intake and muscle mass, treating them as dependent variables with FGF21 as the independent variable. These models were adjusted for the same confounders, with CERI additionally included in the model for protein intake and vice versa. Assumptions of normality were checked via Q–Q plots of residuals.

Cox proportional hazards models were employed to examine associations of FGF21 concentration, protein intake, and CERI with all‐cause mortality. Hazards ratios were presented per doubling of circulating FGF21 concentration and protein intake and per 1 SD increase for CERI. The proportional hazards assumption was visually verified using plots of the scaled Schoenfeld residuals, and this assumption did not violate the models. Cox regression analyses were adjusted for the same potential confounders as used in the logistic regression. Similarly, we also performed stratified analyses according to age groups <65 years or ≥65 years. Furthermore, in separate analyses, we performed additional analyses where FGF21, protein intake, and CERI were adjusted for each other. Moreover, a visual representation of the outcome distribution over time was shown using Kaplan–Meier curves stratified by circulating FGF21 concentration quartiles.

Similar to the analyses of FGF21 and low protein intake, additional sensitivity analyses were conducted by excluding (1) participants with FGF21 levels, protein intake, or CERI in the top or bottom 2.5 percentiles; (2) individuals with type 2 diabetes; (3) those with a BMI outside the range of 18.5–30 kg/m^2^; (4) participants with eGFR < 60 mL/min/1.73 m^2^, and (5) participants with a history of cardiovascular disease. Furthermore, additional models were tested with further adjustments for ketone body concentrations, FLI, hepatic steatosis index, HOMA‐IR, hs‐CRP and GlycA, asthma or COPD, rheumatic disease and a history of malignancy. Lastly, we included sensitivity analyses using cause‐specific mortality.

## Results

### Baseline data

Analyses were performed on 6395 participants (54 ± 12 years and 50% men). The median FGF21 concentration was 896 (540; 1384) pg/mL, median protein intake was 1.01 (0.85; 1.19 g/kg/day, and mean CERI was 4.1 ± 0.9 mmol/day/m^2^. Baseline characteristics are summarized in Table 1, whereas the participant flow throughout the study is depicted in Fig. S1. Combined dot and box plots showing the distributions of FGF21 concentration, protein intake, and muscle mass stratified by age group are shown in Fig. S2. Compared to participants younger than 65 years, those aged 65 years and older had higher FGF21 concentrations (1010 (676; 1542) versus 857 (513; 1335) pg/mL; *p* < 0.001), lower protein intake (0.95 (0.79; 1.11) versus 1.03 (0.86; 1.21) g/kg/day; *p* < 0.001), and lower muscle mass (3.9 ± 0.9 vs. 4.1 ± 0.9 mmol/day/m^2^; *p* < 0.001). Correlations of age with FGF21 concentration, protein intake, and muscle mass are shown in Fig. S3. Higher age was associated with higher FGF21 concentrations (*r* = 0.21; 95% confidence interval [CI]: 0.18 to 0.23; *p* < 0.001) and with lower protein intake (*r* = −0.13; 95% CI: −0.15 to −0.10; *p* < 0.001) and lower muscle mass (*r* = −0.15; 95% CI: −0.17 to −0.12; *p* < 0.001).

**Table 1:** Baseline data in 6395 participants.

FGF21 concentration, median (Q1; Q3), pg/mL	896 (540; 1384)
Protein intake, median (Q1; Q3), g/kg/day	1.01 (0.85; 1.19)
CERI, mean ± SD, mmol/day/m^2^	4.1 ± 0.9
Age, mean ± SD, years	54 ± 12
Sex, *n* (%) male	3168 (50%)
Weight, mean ± SD, kg	80 ± 15
Height, mean ± SD, cm	173 ± 10
BMI, mean ± SD, kg/m^2^	26.8 ± 4.4
Current smokers, *n* (%)	1773 (28%)
Alcohol, *n* (%) with ≥1 consumption/day	1650 (26%)
Diabetes, *n* (%)	372 (6%)
Hypertension, *n* (%)	2116 (34%)
History of cardiovascular disease, *n* (%)	436 (7%)
Plasma cystatin C, mean ± SD, mg/L	0.91 ± 0.21
Plasma creatinine, mean ± SD, µmol/L	73 ± 21
eGFR _CKD‐EPI creatinine − cystatin C 2021_, mean ± SD, mL/min	97 ± 17
Urinary albumin excretion, median (Q1; Q3), mg/day	9 (6; 16)
ALAT, median (Q1; Q3), U/L	17 (13; 24)
ASAT, median (Q1; Q3), U/L	22 (19; 26)
Albumin, median (Q1; Q3), g/L	44 (42; 45)
Total cholesterol, mean ± SD, mmol/L	5.0 ± 0.9
HDL cholesterol, mean ± SD, mmol/L	1.3 ± 0.3
LDL cholesterol, mean ± SD, mmol/L	3.0 ± 0.8
Triglycerides, median (Q1; Q3), mmol/L	1.1 (0.8; 1.6)
Glucose, median (Q1; Q3), mmol/L	4.8 (4.4; 5.3)
Insulin, median (Q1; Q3), mU/L	8.3 (5.8; 12.3)
GlycA, mean ± SD, µmol/L	380 ± 61
hs‐CRP, median (Q1; Q3), mg/L	1.4 (0.6; 3.1)
HOMA‐IR, median (Q1; Q3), (mU/L^2^)/22.5	1.7 (1.2; 2.8)
Ketone bodies, median (Q1; Q3), µmol/L	177 (139; 248)

Abbreviations: ALAT, alanine‐aminotransferase; ASAT, aspartate aminotransferase; BMI, body mass index; CERI, creatinine excretion rate index; CKD‐EPI, chronic kidney disease epidemiology collaboration; FGF21, fibroblast growth factor 21; GlycA, glycoprotein acetylation; HDL, high‐density lipoprotein; HOMA‐IR, homeostatic model assessment for insulin resistance; hs‐CRP, high‐sensitivity C‐reactive protein; LDL, low‐density lipoprotein.

### Logistic regression analyses of the association between circulating FGF21 and low protein intake

**Fig. 1:**
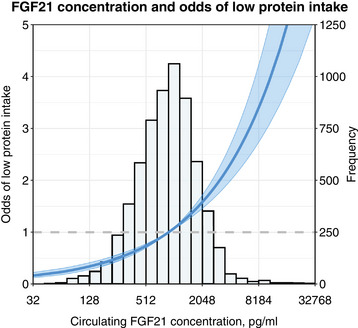
Graphical depiction of the association between circulating FGF21 concentration and low protein intake (<0.8 g/kg/day). Analyses were adjusted for age, sex, BMI, eGFR, urinary albumin excretion, smoking status, alcohol consumption, hypertension, diabetes, history of cardiovascular disease, HDL cholesterol, LDL cholesterol, triglycerides, and plasma albumin concentration. BMI, body mass index; CERI, creatinine excretion rate index; FGF21, fibroblast growth factor 21; HDL, high‐density lipoprotein; LDL, low‐density lipoprotein.

Among the study population, 1242 participants (19%) had a protein intake below 0.8 g/kg/day, classified as low protein intake. Results of the logistic regression analysis examining the association between FGF21 and low protein intake are presented in Table 2. In unadjusted analyses, higher FGF21 concentrations were linked to an increased odds of low protein intake (odds ratio [OR] [95% CI]: 1.56 [1.47–1.67]; *p* < 0.001). This association remained statistically significant after adjustment for potential confounders (OR: 1.48 [1.38–1.58]; *p* < 0.0001). A graphical representation of this relationship is provided in Fig. 1. Stratified analyses across age groups (<65 vs. ≥65 years) are shown in Table S1. In sensitivity analyses after excluding outliers, participants with diabetes, participants with low or high BMI, participants with impaired kidney function, and participants without a history of cardiovascular disease (Table S2), similar point estimates were found. In additional sensitivity analyses, the association was also unaltered after adjusting for either CERI, ketones, FLI, HSI, HOMA‐IR, hs‐CRP and GlycA, asthma or COPD, rheumatic disease, and a history of malignancy (Table S3). Lastly, when using alternative cut‐offs for low protein intake, 0.6 or 1.0 g/kg/day (Table S4), elevated FGF21 concentrations continued to show a significant association with a higher odds of low protein intake, with ORs (95% CI) of 1.64 (1.45; 1.87) and 1.35 (1.29; 1.43), respectively (*p* < 0.0001 for both).

**Table 2:** Logistic regression analyses of the association between circulating FGF21 and low protein intake.

	<0.8 g/day/kg	
Model	OR (95% CI)	*p* value
Model 1	1.56 (1.47; 1.67)	<0.0001
Model 2	1.52 (1.43; 1.62)	<0.0001
Model 3	1.47 (1.38; 1.57)	<0.0001
Model 4	1.48 (1.38; 1.58)	<0.0001
*Events, n (%)*	*1242 (19%)*	

*Note*: FGF21 is the independent variable in the analyses. Model 1: Crude. Model 2: Adjusted for age and sex. Model 3: Further adjusted for BMI, eGFR, and urinary albumin excretion. Model 4: Additionally adjusted for smoking, alcohol intake, hypertension, diabetes, history of cardiovascular disease, HDL cholesterol, LDL cholesterol, triglycerides, and plasma albumin concentration.

Abbreviations: BMI, body mass index; CI, confidence interval; eGFR, estimated glomerular filtration rate; FGF21, fibroblast growth factor 21; HDL, high‐density lipoprotein; LDL, low‐density lipoprotein; OR, odds ratio.

### Linear regression analyses of the association between FGF21 concentration, protein intake, and muscle mass

Linear regression analyses examining FGF21 concentration as the independent variable with protein intake and CERI as dependent variables are presented in Table 3. Univariably, a higher FGF21 concentration was associated with a lower protein intake (standardized beta [Std. β] (95% CI): −0.25 (−0.28; −0.23); *p* < 0.0001) and to a lesser degree lower CERI (Std. β (95% CI): −0.03 (−0.05; −0.01); *p* = 0.034). The associations of higher FGF21 with lower protein (Std. β (95% CI): −0.19 (−0.21; −0.16); *p* < 0.0001) and CERI (Std. β (95% CI): −0.08 (−0.10; −0.06); *p* < 0.0001) were independent of the potential confounders. After adjusting for CERI, the association of FGF21 with protein intake weakened slightly but remained significant (Std. β (95% CI): −0.15 (−0.17; −0.13); *p* < 0.0001). In contrast, after adjusting for protein intake, the association of FGF21 with CERI lost significance (Std. β (95% CI): −0.01 (−0.03; 0.01); *p* = 0.49).

**Table 3:** Linear regression analyses on the association of circulating FGF21 with protein intake and measures of muscle mass.

	Protein intake		Muscle mass by CERI	
Model	Std. β (95% CI)	*p* value	Std. β (95% CI)	*p* value
Model 1	−0.25 (−0.28; −0.23)	<0.0001	−0.03 (−0.05; −0.01)	0.034
Model 2	−0.24 (−0.26; −0.21)	<0.0001	−0.07 (−0.10; −0.05)	<0.001
Model 3	−0.20 (−0.22; −0.18)	<0.0001	−0.08 (−0.10; −0.06)	<0.001
Model 4	−0.19 (−0.21; −0.16)	<0.0001	−0.08 (−0.10; −0.06)	<0.001
Model 5	−0.15 (−0.17; −0.13)	<0.0001	−0.01 (−0.03; 0.01)	0.49

*Note*: Protein intake and muscle mass are the dependent variables, and FGF21 is the independent variable in the linear regression analyses. Model 1: Crude. Model 2: Adjusted for age, sex, and BMI. Model 3: Further adjusted for eGFR and urinary albumin excretion. Model 4: Extended with adjustments for smoking, alcohol intake, hypertension, diabetes, history of cardiovascular disease, HDL cholesterol, LDL cholesterol, triglycerides, and plasma albumin concentration. Model 5: Additionally adjusted for protein intake (analyses of CERI) or CERI (analyses of protein intake).

Abbreviations: BMI, body mass index; CERI, creatinine excretion rate index; CI, confidence interval; eGFR, estimated glomerular filtration rate; FGF21, fibroblast growth factor 21; HDL, high‐density lipoprotein; LDL, low‐density lipoprotein; Std. β, standardized beta.

### Cox regression of FGF21 concentration, protein intake, and muscle mass with all‐cause mortality

**Fig. 2:**
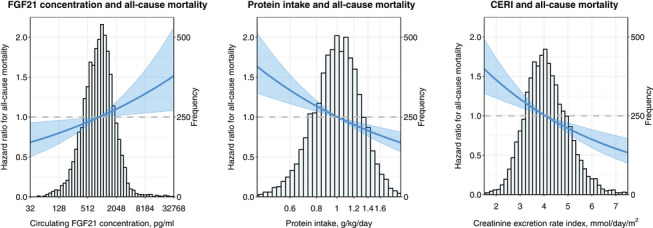
Graphical depiction of the association between circulating FGF21 levels, protein intake, and CERI with all‐cause mortality. Analyses were adjusted for age, sex, BMI, eGFR, urinary albumin excretion, smoking status, alcohol consumption, hypertension, diabetes, history of cardiovascular disease, HDL cholesterol, LDL cholesterol, triglycerides, and plasma albumin concentration. BMI, body mass index; CERI, creatinine excretion rate index; eGFR, estimated glomerular filtration rate; FGF21, fibroblast growth factor 21; HDL, high‐density lipoprotein; LDL, low‐density lipoprotein.

During a median follow‐up of 10.4 (8.3; 14.3) years, a total of 955 (15%) of the participants died. Participants who died had higher baseline FGF21 concentration (1047 (690; 1666) vs. 866 (522; 1213) pg/mL; *p* < 0.0001) and lower baseline protein intake (0.95 (0.79; 1.10) vs. 1.02 (0.86; 1.20) g/kg/day; *p* < 0.0001) and lower CERI (3.9 ± 0.9 vs. 4.2 ± 0.9 mmol/day/m^2^) as compared to participants who remained alive during follow‐up. Kaplan–Meier survival curves for quartiles of plasma FGF21 concentration are shown in Fig. S4, indicating an association between higher FGF21 concentration and increased all‐cause mortality over time (log‐rank test *p* < 0.001). A summary of the Cox regression analyses is provided in Table 4. Univariably, a higher FGF21 concentration was associated with a higher risk of all‐cause mortality (hazard ratio (HR) per doubling (95% CI): 1.27 (1.20; 1.34); *p* < 0.0001). After adjusting for potential confounders, the association weakened but remained significant (HR (95% CI): 1.09 (1.02; 1.16); *p* = 0.009). Univariably, a higher protein intake (HR per doubling (95% CI): 0.50 (0.43; 0.58); <0.0001) and higher CERI (HR per SD increase (95% CI): 0.79 (0.74; 0.84); *p* < 0.0001) were associated with a reduced risk of mortality. The associations of higher protein intake (HR (95% CI): 0.67 (0.56; 0.81); *p* < 0.0001) and higher CERI (HR (95% CI): 0.83 (0.76; 0.90); *p* < 0.0001) with a lower risk of all‐cause mortality remained significant after adjusting for potential confounders. A graphical representation of the associations of FGF21 concentration, protein intake, and CERI with all‐cause mortality is shown in Fig. 2. Stratified analyses across age groups are shown in Table S5 and demonstrated that the association of protein intake is more pronounced in elderly (≥65 years).

**Table 4:** Cox regression analyses with all‐cause mortality.

	FGF21 concentrations		Protein intake		Muscle mass	
Model	HR (95% CI)	*p* value	HR (95% CI)	*p* value	HR (95% CI)	*p* value
Model 1	1.27 (1.20; 1.34)	<0.0001	0.50 (0.43; 0.58)	<0.0001	0.79 (0.74; 0.84)	<0.0001
Model 2	1.17 (1.10; 1.24)	<0.0001	0.63 (0.53; 0.75)	<0.0001	0.82 (0.76; 0.89)	<0.0001
Model 3	1.12 (1.05; 1.19)	0.0004	0.59 (0.50; 0.71)	<0.0001	0.78 (0.72; 0.85)	<0.0001
Model 4	1.09 (1.02; 1.16)	0.009	0.67 (0.56; 0.81)	<0.0001	0.83 (0.76; 0.90)	<0.0001

*Note*: There were in total 955 (15%) deaths during follow‐up. HR is presented per doubling for FGF21 and protein intake and per SD deviation increase for muscle mass. Model 1: Crude. Model 2: Adjusted for age, sex, and BMI. Model 3: Further adjusted for eGFR and urinary albumin excretion. Model 4: Additionally adjusted for smoking, alcohol intake, hypertension, diabetes, history of cardiovascular disease, HDL cholesterol, LDL cholesterol, triglycerides, and plasma albumin concentration. Linearity was assessed by comparing the fit of a linear model to that of a non‐linear model using an analysis of variance test. The non‐linear model included a natural spline with 2 degrees of freedom. A significant improvement in model fit was interpreted as evidence against linearity.

Abbreviations: BMI, body mass index; CI, confidence interval; eGFR, estimated glomerular filtration rate; FGF21, fibroblast growth factor 21; HDL, high‐density lipoprotein; HR, hazard ratio; LDL, low‐density lipoprotein.

Sensitivity analyses after excluding participants with outliers in the primary variables, with diabetes, with high or low BMI, with low eGFR or with a history of cardiovascular disease are shown in Table S6. The associations of FGF21 were all robust in all sensitivity analyses in which subpopulations were investigated, with the exceptions of the association of FGF21 concentration with all‐cause mortality after excluding participants with lowest and highest 2.5 percentile of CERI and after excluding participants with a history of cardiovascular disease. The associations of protein intake and CERI with all‐cause mortality were robust in all sensitivity analyses where subpopulations were investigated.

Sensitivity analyses with additional adjustments for ketone bodies, FLI, HSI, HOMA‐IR, the inflammation markers GlycA and hs‐CRP, and comorbidities asthma or COPD, rheumatic disease, and a history of malignancy are shown in Table S7. The associations of FGF21 concentration, protein intake, and CERI with all‐cause mortality remained significant in all these analyses. Analyses of FGF21 concentration, protein intake, and CERI with cause‐specific mortality are shown in Table S8. FGF21 concentration and protein intake were primarily associated with non‐cardiovascular mortality, whereas CERI was associated with both cardiovascular and non‐cardiovascular mortality.

To investigate the interrelations of the associations of FGF21 concentration, protein intake, and CERI with mortality, separate analyses where the associations were stepwise adjusted for each other are shown in Table 5. These analyses demonstrate that the association of FGF21 with all‐cause mortality was weakened and lost significance after adjusting for protein intake, but not after additional adjustment for CERI, whereas the associations of protein intake and CERI remained largely unchanged after adjusting for FGF21 concentration. The association of protein intake was slightly weakened but remained significant after adjusting for CERI.

**Table 5:** Sensitivity analyses of the analyses with mortality with additional adjustments for each other and muscle mass.

	FGF21 concentrations		Protein intake		CERI	
Model	HR (95% CI)	*p* value	HR (95% CI)	*p* value	HR (95% CI)	*p* value
Base	1.09 (1.02; 1.16)	0.009	0.67 (0.56; 0.81)	<0.0001	0.83 (0.76; 0.90)	<0.0001
+FGF21	—	—	0.69 (0.58; 0.83)	<0.0001	0.83 (0.77; 0.91)	<0.0001
+Protein intake	1.06 (0.99; 1.14)	0.089	—	—	0.88 (0.80; 0.97)	0.010
+CERI	1.08 (1.01; 1.15)	0.019	0.78 (0.63; 0.96)	0.018	—	—

*Note*: HR is presented per doubling for FGF21 and protein intake and per SD deviation increase for muscle mass. Base model is adjusted for age and sex, BMI, eGFR and urinary albumin excretion, smoking, alcohol intake, hypertension, diabetes, history of cardiovascular disease, HDL cholesterol, LDL cholesterol, triglycerides, and plasma albumin concentration.

Abbreviations: BMI, body mass index; CI, confidence interval; eGFR, estimated glomerular filtration rate; FGF21, fibroblast growth factor 21; HDL, high‐density lipoprotein; HR, hazard ratio; LDL, low‐density lipoprotein.

## Discussion

In this observational cohort study, a higher circulating FGF21 concentration was associated with higher odds of having a low protein intake, regardless of the cut‐off used. Furthermore, a higher FGF21 concentrations appeared to be associated with a low muscle mass; however, this association was driven by the relationship with protein intake. Lastly, elevated FGF21, lower protein intake, and lower muscle mass were associated with increased risk of mortality. Lower protein intake and lower muscle mass were associated with increased risk of mortality, independent of each other. The association of FGF21 with increased risk of mortality was confounded by protein intake.

FGF21 is a liver‐derived metabolic hormone that plays a central role in the adaptive response to protein restriction. It signals via FGFR1c and its co‐receptor β‐Klotho and is believed to regulate protein appetite and macronutrient selection [26, 27]. Animal studies have shown that FGF21 increases protein intake and shifts food preferences under conditions of low protein availability, supporting its role in maintaining protein homeostasis [1, 28–32]. This aligns with the *protein leverage hypothesis*, which posits that organisms prioritize protein intake, often at the expense of excess energy consumption when dietary protein content is limited [26, 33, 34]. Although much of the mechanistic evidence derives from rodent models, recent human data suggest that protein intake is a major driver of circulating FGF21 concentrations [26, 33, 34]. Our finding that higher FGF21 concentrations are associated with lower protein intake supports the view that chronically low protein availability triggers FGF21 secretion in humans.

Experimental studies in mice have shown that FGF21 plays a key role in regulating protein‐specific appetite through central mechanisms [26]. Exogenous administration of FGF21 increases protein intake in rodents, whereas mice lacking FGF21 or brain‐specific β‐Klotho do not increase food intake when given a low‐protein diet. [26]. Elevated FGF21 levels have also been associated with reduced sweet preference, suggesting a broader role in dietary modulation [26]. Protein intake appears to be the primary regulator of circulating FGF21 levels, especially when carbohydrate intake is high [27]. Supporting translation to humans, a recent study in humans found that protein intake has a stronger influence on FGF21 secretion than sugar [35].

Previous studies have shown that FGF21 concentrations increase with age, whereas protein intake and muscle mass decline [36, 37]. Consistent with these findings, we observed higher FGF21 concentrations and lower protein intake and muscle mass in older participants, both when comparing age strata (<65 vs. ≥65 years) and in continuous analyses. Furthermore, we also found that the associations of protein intake with mortality were more pronounced in the elderly, highlighting the importance of protein intake in the elderly.

Elevated FGF21 concentrations are also observed in individuals with metabolic disorders, such as type 2 diabetes and obesity, who often exhibit FGF21 resistance—a reduced biological response to FGF21 despite its increased levels [12, 38]. It is conceivable that a similar resistance develops in the context of chronic protein deficiency, where persistently elevated FGF21 fails to restore protein homeostasis. This may further impair metabolic adaptation, promote muscle catabolism, and contribute to adverse outcomes.

Increased FGF21 concentrations have been linked to a higher mortality risk in patients with end‐stage kidney disease [6], hemodialysis patients [39], heart failure [40], and coronary artery disease [5]. Recently, Tucker et al. demonstrated an association between elevated FGF21 concentrations and a higher risk of all‐cause mortality [7]. This relationship was independent of several confounders; however, their study did not include data on dietary protein intake, a key variable that our study integrates into the analyses. Consistent with findings from Tucker's study, our results also demonstrated that elevated FGF21 levels were linked to a higher risk of mortality. In the liver, FGF21 is directly upregulated by PPARα as a response to fasting, aligning with its role as a metabolic regulator during nutrient scarcity. In our study, participants were instructed to fast overnight before plasma collection, minimizing variability due to recent food intake. To further account for fasting effects, we measured circulating ketone bodies as a proxy for the degree of fasting. Notably, accounting for the effect of ketone body concentrations did not materially affect the point estimates of the associations of FGF21 with either low protein intake or all‐cause mortality. Elevated FGF21 concentrations are also observed in MAFLD, a condition independently linked to higher mortality risk [41, 42]. To disentangle the confounding effects of liver disease, we adjusted for proxies of MAFLD, including the fatty liver and hepatic steatosis indices. Despite these adjustments, the point estimates of the associations of FGF21 with low protein intake and mortality were similar. Likewise, adjustment for systemic inflammation, a known driver of both elevated FGF21 and adverse health outcomes, by accounting for hs‐CRP and GlycA did not materially affect the point estimates [43].

Additionally, higher circulating FGF21 concentrations have been reported in individuals with insulin resistance and type 2 diabetes [12]. Consistent with this, we previously demonstrated that higher FGF21 concentrations predict an increased risk of diabetes [12]. Despite this established link, the associations of FGF21 with low protein intake and all‐cause mortality in our study were independent of adjustments for diabetes status and HOMA‐IR in sensitivity analyses. Analyses excluding participants with type 2 diabetes yielded comparable results. Strikingly, only adjustments for protein intake itself abrogated the association of FGF21 with all‐cause mortality. The loss of the association between FGF21 and all‐cause mortality after adjusting for protein intake likely reflects the primary role of dietary protein deficiency in driving FGF21 secretion. Elevated FGF21 concentrations signal adaptive responses to low protein availability, and their prognostic value for mortality may largely stem from the underlying nutritional state they represent. Once dietary protein intake is accounted for, the residual impact of FGF21 on mortality diminishes, suggesting that it is not FGF21 per se but rather the nutritional deficit to which it is linked, which is the principal determinant of mortality risk. This interpretation aligns with the observed role of protein intake in maintaining metabolic and physiological resilience. Chronic protein deficiency exacerbates catabolic processes, reduces muscle mass, and impairs immune function, collectively increasing vulnerability to adverse outcomes. In our study, we found that lower protein intake was associated with a higher risk of mortality. Interestingly, the point estimates of the association of protein intake with all‐cause mortality lowered slightly after adjusting for muscle mass, indicating that this association may be partly, but not fully, explained by lower muscle mass.

The question of whether a higher or lower protein intake is beneficial in the general population has been long debated. A meta‐analysis examining the relationship between total protein intake and all‐cause mortality reported that, among 247,863 participants, 59,841 deaths occurred during a follow‐up of 12–28 years; higher protein intake was linked to an increased risk of all‐cause mortality (risk ratio for highest vs. lowest intake category: 1.05, 95% CI: 1.01–1.10). In contrast, a meta‐analysis that comprised 21 prospective cohort studies found that, among 480,304 participants and 72,261 deaths in 3.5–32 years follow‐up, higher protein intake was associated with a lower risk of mortality (risk ratio for highest vs. lowest intake category: 0.94, 95% CI: 0.89–0.99). To be noted, in these meta‐analyses, most of the studies that were considered used classic dietary assessments, and only a few used biomarker‐based methods. One single center study found that low urinary urea nitrogen excretion, defined as a threshold value below which 20% of urinary urea nitrogen excretion values in the study sample fell, was associated with a higher risk of mortality in 4679 adults from the Gubbio cohort in Italy (HR 1.31, 95% CI 1.12–1.53) over an average 16‐year follow‐up [44]. In another study, Courand et al. studied 1128 patients with hypertension but no chronic kidney disease for 10 years to evaluate the impact of protein intake on mortality. After adjustments, patients with a higher protein intake had a reduced risk of all‐cause mortality (HR 0.65 (0.43–0.98)) [45]. A biomarker‐based approach overcomes several limitations of traditional dietary assessment methods, such as literacy and motivation constraints, over or underestimating intake, inaccuracies in portion size estimation, dietary alterations due to self‐monitoring, and the tendency to provide a socially desirable response [46, 47, 48, 49]. Moreover, dietary diaries do not provide insights into bioavailability. On the other hand, the biomarker‐based method does not allow for differentiation between animal and plant protein, which likely influence the associations with mortality. Furthermore, the biomarker method does not provide data on total energy intake. This has prevented us from drawing definitive conclusions.

This study also demonstrated an association between higher FGF21 concentrations and lower muscle mass. Oost et al. previously demonstrated that FGF21 drives muscle atrophy under metabolic stress, such as fasting, by enhancing mitophagy through the mitophagy protein Bnip3 and reducing protein synthesis [50]. In FGF21 knockout mice, fasting‐induced muscle loss was attenuated due to preserved protein synthesis and reduced mitochondrial degradation [50]. The attenuation of the association between FGF21 and muscle mass in our study after adjusting for protein intake highlights the importance of dietary protein in mitigating catabolic effects related to FGF21 [50]. In line with previous literature, we also found that higher muscle mass is associated with reduced risk of all‐cause mortality. In a meta‐analysis of 49 studies, Zhou et al. demonstrated that muscle wasting was linked to an increased risk of all‐cause mortality in the general population (RR = 1.36, 95% CI, 1.28–1.44) [8]. Furthermore, low muscle mass as a continuous variable has also been linked to a higher risk of mortality in the NHANES study [51], the Health Professionals Follow‐up Study [52], and also previously in the PREVEND study [53].

Key strengths of this study include the long and comprehensive follow‐up period, along with extensive data collection, enabling adjustment for a broad range of confounders. A limitation of this study is that the data were collected in the early 2000s, when obesity, type 2 diabetes, physical inactivity, and the consumption of ultra‐processed foods were less prevalent than today. These secular changes could have potentially influenced the relationship between FGF21 concentration, protein intake, muscle mass, and mortality. Another limitation of the study is the lack of data on physical activity levels, which is an important potential confounder, as physical activity influences both muscle mass and metabolic pathways associated with FGF21 and protein intake. Information on comorbidities was primarily obtained through self‐reported questionnaires, and we were unable to access medical records. As a result, complete data on all potentially confounding comorbidities (e.g., arrhythmias, valvular disease, and dementia) were not available. Furthermore, the observational design prevents us from drawing conclusions on whether the found associations are causally or merely associative. Lastly, we cannot elucidate the underlying mechanisms driving these associations.

In conclusion, this study demonstrated that elevated circulating FGF21 concentrations are associated with low protein intake in a large cohort of the general population. Furthermore, a higher FGF21 concentration, lower protein intake, and lower muscle mass were each associated with increased risk of all‐cause mortality. The associations of FGF21 with mortality appears to be based on its function as a marker for low protein intake, as the association lost significance after adjusting for protein intake. Integrating FGF21 as a biomarker in clinical practice warrants further investigation. Its utility in detecting individuals at heightened risk of protein deficiency or adverse outcomes, particularly in the context of aging or chronic disease, could help guide preventive strategies and improve clinical outcomes.

## Author contributions

**Adrian Post**: Investigation; conceptualization; methodology; writing—original draft. **Wendy A. Dam**: Data curation; writing—review and editing. **Dion Groothof**: Writing—review and editing. **Casper F. M. Franssen**: Writing—review and editing. **Stephan J. L. Bakker**: Writing—review and editing; supervision; funding acquisition; resources. **Robin P. F. Dullaart**: Supervision; writing—original draft; methodology; conceptualization.

## Conflict of interest statement

The authors declare no conflicts of interest.

## Funding information

None.

## Supporting information




**Figure S1**. Participant flow through the study.
**Figure S2**. Combined dot and box plots showing the distributions of FGF21 concentration, protein intake, and muscle mass in participants of the PREVEND cohort, stratified by age group. 5011 participants were younger than 65 years, and 1384 participants were 65 years or older. The geom_quasirandom function in R was used to offset individual data points and reduce overplotting. Asterisks (***) indicate statistically significant differences between age groups (*p* < 0.001). Compared to participants younger than 65 years, those aged 65 years and older had higher FGF21 concentrations (1010 (676; 1542) vs. 857 (513; 1335) pg/mL; *p* < 0.001), lower protein intake (0.95 (0.79; 1.11) vs. 1.03 (0.86; 1.21) g/kg/day; *p* < 0.001), and lower muscle mass (3.9 ± 0.9 vs. 4.1 ± 0.9 mmol/day/m^2^; *p* < 0.001).
**Figure S3**. Scatterplots with linear regression lines showing the associations of age with FGF21 concentration (A), protein intake (B), and muscle mass (C) in participants of the PREVEND cohort. Higher age was associated with higher FGF21 concentrations (*r* = 0.21; 95% CI: 0.18–0.23; *p* < 0.001) and with lower protein intake (*r* = −0.13; 95% CI: −0.15 to −0.10; *p* < 0.001) and lower muscle mass (*r* = −0.15; 95% CI: −0.17 to −0.12; *p* < 0.001).
**Figure S4**. Kaplan–Meier survival curves for quartiles of plasma FGF21 levels. The plot displays all‐cause mortality stratified by quartiles of circulating FGF21 concentration, with quartile 1 representing the lowest quartile and quartile 4 the highest. The survival probability decreases over time across all quartiles, with a significant difference observed between groups (log‐rank test, *p* < 0.001), indicating higher FGF21 levels are associated with a higher risk of mortality.
**Table S1**. Sensitivity analyses of the logistic regression analyses of FGF21 concentration with low protein intake (<0.8 g/day/kg) according to age <65 years or ≥65 years.
**Table S2**. Sensitivity analyses on the association of FGF21 concentration with low protein intake in various subpopulations.
**Table S3**. Sensitivity analyses on the association of FGF21 concentration with low protein intake using additional adjustments.
**Table S4**. Logistic regression analyses of circulating FGF21 with low protein intake using alternative cutoffs,
**Table S5**. Cox regression analyses with all‐cause mortality according to age subgroups.
**Table S6**. Sensitivity analyses on the association of circulating FGF21 concentration, protein intake, and CERI with all‐cause mortality in selected subgroups.
**Table S7**. Sensitivity analyses on the association of circulating FGF21 with all‐cause mortality with additional adjustments.
**Table S8**. Sensitivity analyses on the association of circulating FGF21 with all‐cause mortality with cause‐specific mortality.

## Data Availability

The data, codebook, and analytical code referenced in this manuscript will be provided upon request from the editor.
