# Supplementary material for: Higher circulating FGF21, lower protein intake, and lower muscle mass: Associations with a higher risk of mortality
Source: J Intern Med. 2025 May 19;298(1):2–15. doi: 10.1111/joim.20099 (PMC12159714; doi:10.1111/joim.20099)
Supplement: Supplementary file 1 — Figure S1. Participant flow through the study. Figure S2. Combined dot and box plots showing the distributions of FGF21 concentration, protein intake, and muscle mass in participants of the PREVEND cohort, stratified by age group. 5011 participants were younger than 65 years, and 1384 participants were 65 years or older. The geom_quasirandom function in R was used to offset individual data points and reduce overplotting. Asterisks (***) indicate statistically significant differences between age groups (p < 0.001). Compared to participants younger than 65 years, those aged 65 years and older had higher FGF21 concentrations (1010 (676; 1542) vs. 857 (513; 1335) pg/mL; p < 0.001), lower protein intake (0.95 (0.79; 1.11) vs. 1.03 (0.86; 1.21) g/kg/day; p < 0.001), and lower muscle mass (3.9 ± 0.9 vs. 4.1 ± 0.9 mmol/day/m2; p < 0.001). Figure S3. Scatterplots with linear regression lines showing the associations of age with FGF21 concentration (A), protein intake (B), and muscle mass (C) in participants of the PREVEND cohort. Higher age was associated with higher FGF21 concentrations (r = 0.21; 95% CI: 0.18–0.23; p < 0.001) and with lower protein intake (r = −0.13; 95% CI: −0.15 to −0.10; p < 0.001) and lower muscle mass (r = −0.15; 95% CI: −0.17 to −0.12; p < 0.001). Figure S4. Kaplan–Meier survival curves for quartiles of plasma FGF21 levels. The plot displays all‐cause mortality stratified by quartiles of circulating FGF21 concentration, with quartile 1 representing the lowest quartile and quartile 4 the highest. The survival probability decreases over time across all quartiles, with a significant difference observed between groups (log‐rank test, p < 0.001), indicating higher FGF21 levels are associated with a higher risk of mortality. Table S1. Sensitivity analyses of the logistic regression analyses of FGF21 concentration with low protein intake (<0.8 g/day/kg) according to age <65 years or ≥65 years. Table S2. Sensitivity analyses on the association of FGF21 conce [file JOIM-298-2-s001.docx]

**Supplementary data to “Higher circulating FGF21, lower protein intake, and lower muscle mass: Associations with a higher risk of mortality”**


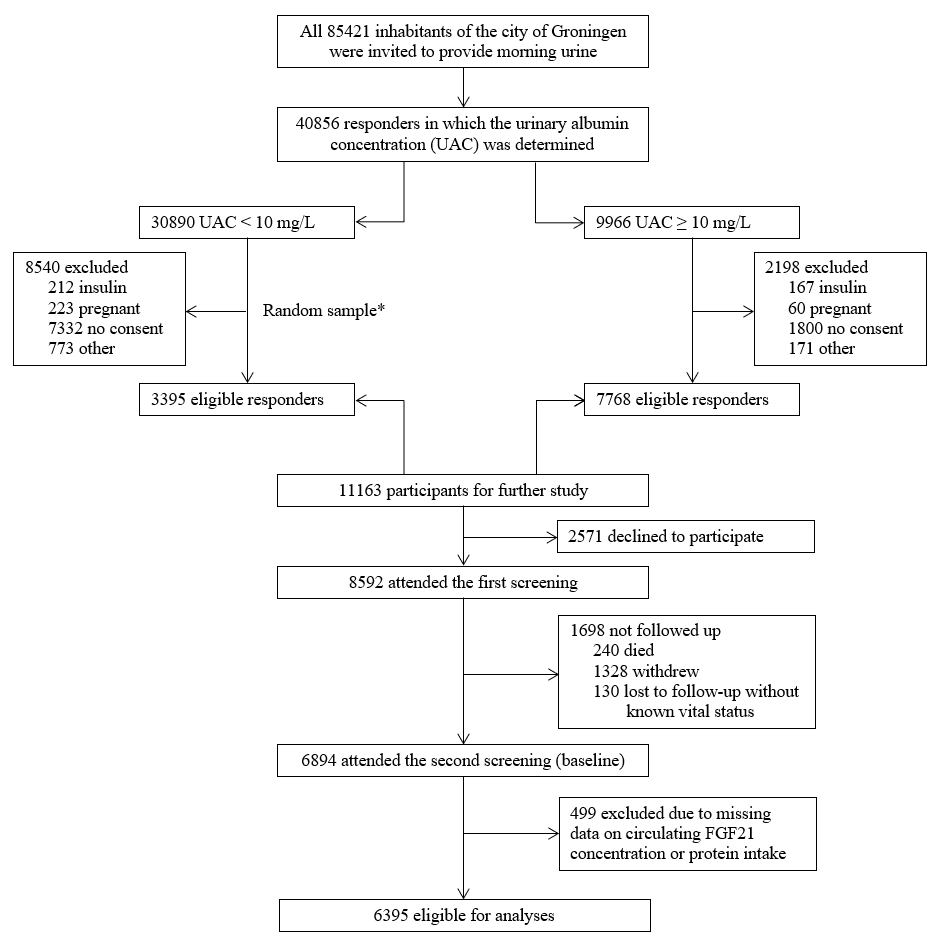


**Figure S1.** Participant flow through the study.


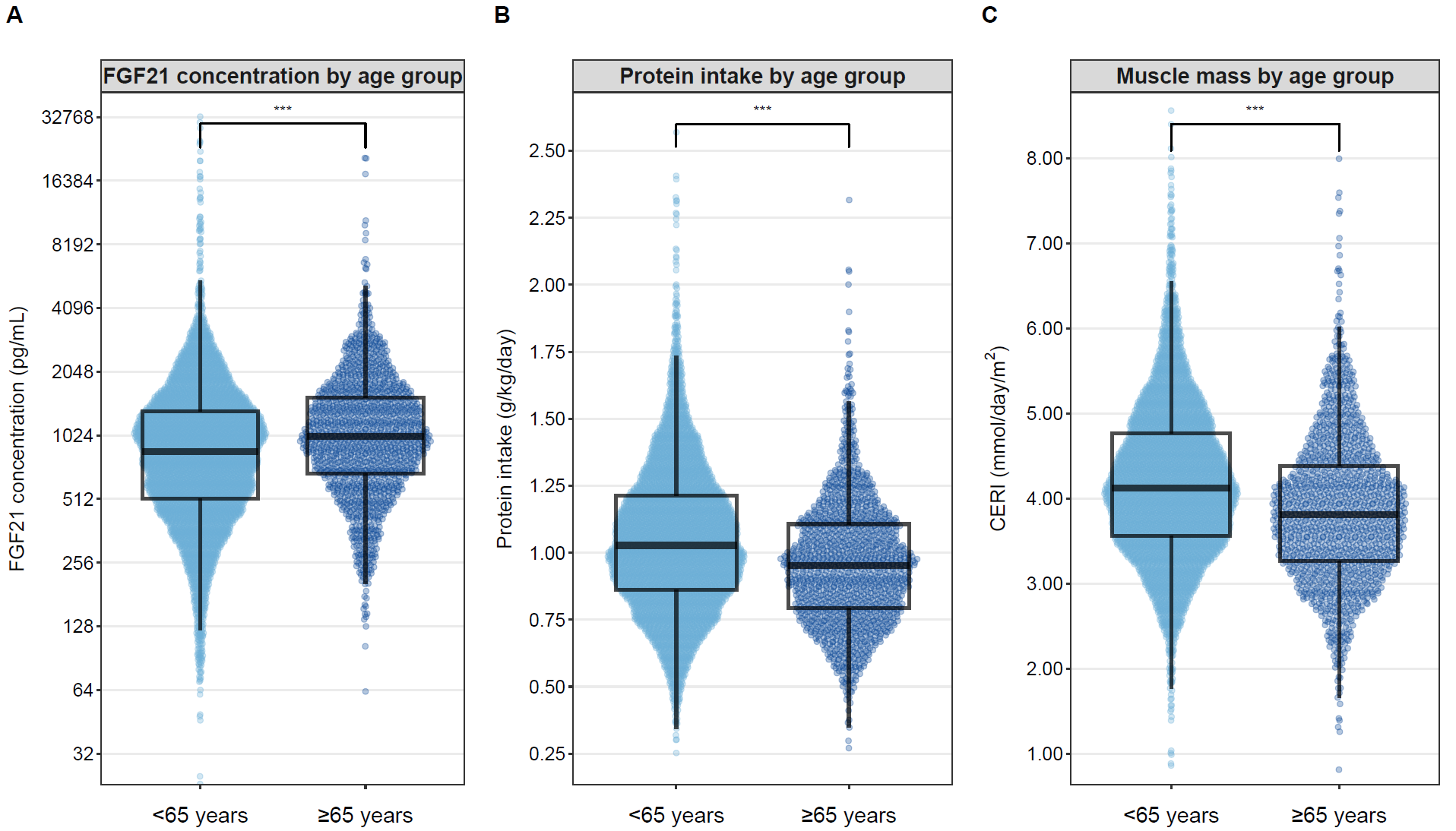


**Figure S2.** Combined dot and box plots showing the distributions of FGF21 concentration, protein intake, and muscle mass in participants of the PREVEND cohort, stratified by age group. 5011 participants were younger than 65 years and 1384 participants were 65 years or older. The geom_quasirandom function in R was used to offset individual data points and reduce overplotting. Asterisks (***) indicate statistically significant differences between age groups (P < 0.001). Compared to participants younger than 65 years, those aged 65 years and older had higher FGF21 concentrations (1010 (676; 1542) versus 857 (513; 1335) pg/mL; P<0.001), lower protein intake (0.95 (0.79; 1.11) versus 1.03 (0.86; 1.21) g/kg/day; P<0.001), and lower muscle mass (3.9 ± 0.9 versus 4.1 ± 0.9 mmol/day/m^2^; P<0.001).


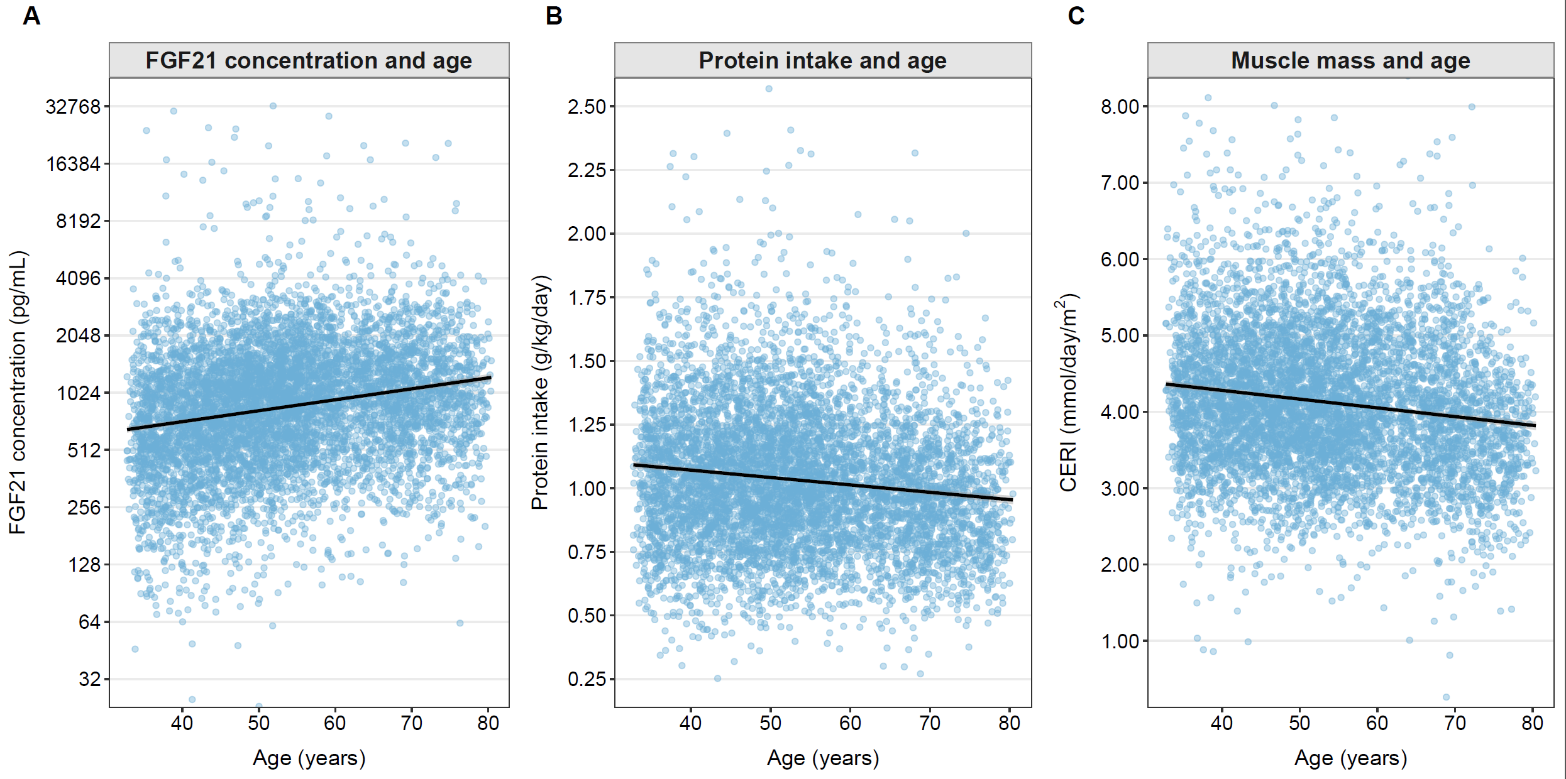


**Figure S3.** Scatterplots with linear regression lines showing the associations of age with FGF21 concentration (A), protein intake (B), and muscle mass (C) in participants of the PREVEND cohort. Higher age was associated with higher FGF21 concentrations (r = 0.21; 95% CI: 0.18 to 0.23; *P* < 0.001), and with lower protein intake (r = –0.13; 95% CI: –0.15 to –0.10; *P* < 0.001) and lower muscle mass (r = –0.15; 95% CI: –0.17 to –0.12; *P* < 0.001).

| **Table S1.** Sensitivity analyses of the logistic regression analyses of FGF21 concentration with low protein intake (<0.8 g/day/kg) according to age <65 years or ≥ 65 years | | | | |
| --- | --- | --- | --- | --- |
|  | **Age < 65 years** | | **Age ≥ 65 years** | |
| **Model** | **OR (95% CI)** | **P-value** | **OR (95% CI)** | **P-value** |
| Model 1 | 1.55 (1.44; 1.66) | <0.0001 | 1.51 (1.32; 1.72) | <0.0001 |
| Model 2 | 1.58 (1.48; 1.70) | <0.0001 | 1.47 (1.29; 1.68) | <0.0001 |
| Model 3 | 1.54 (1.44; 1.67) | <0.0001 | 1.38 (1.21; 1.59) | <0.0001 |
| Model 4 | 1.50 (1.38; 1.62) | <0.0001 | 1.42 (1.23; 1.65) | <0.0001 |
| Odds ratios are presented per doubling of FGF21 concentration.  Model 1: Crude  Model 2: Adjusted for age and sex  Model 3: Further adjusted for BMI, eGFR and urinary albumin excretion.  Model 4: Additionally adjusted for smoking, alcohol intake, hypertension, diabetes, history of cardiovascular disease, HDL cholesterol, LDL cholesterol, triglycerides, plasma albumin concentration.  Abbreviations: BMI: body mass index; CI: confidence interval; eGFR: estimated glomerular filtration rate; FGF21: fibroblast growth factor 21; HDL: high-density lipoprotein; HR: hazard ratio; LDL: low-density lipoprotein;  FGF21: fibroblast growth factor 21; OR: odds ratio; CI: confidence interval; BMI: body mass index; eGFR: estimated glomerular filtration rate; HDL: high-density lipoprotein; LDL: low-density lipoprotein | | | | |

| **Table S2.** Sensitivity analyses on the association of FGF21 concentration with low protein intake in various subpopulations. | | | |
| --- | --- | --- | --- |
|  | **Low protein intake defined as <0.8 g/day/kg** | | |
| **Model** | **Sample size** | **OR (95% CI)** | **P-value** |
| Full population | 6395 | 1.48 (1.38; 1.58) | <0.0001 |
| No outliers FGF21 | 6075 | 1.49 (1.37; 1.63) | <0.0001 |
| No participants with diabetes | 6023 | 1.48 (1.38; 1.59) | <0.0001 |
| No BMI <18.5 kg/m^2^ | 6363 | 1.46 (1.37; 1.57) | <0.0001 |
| No BMI >30 kg/m^2^ | 5154 | 1.48 (1.37; 1.60) | <0.0001 |
| No eGFR <60 ml/min/1.73m^2^ | 5883 | 1.48 (1.38; 1.60) | <0.0001 |
| No history of cardiovascular disease | 5959 | 1.49 (1.39; 1.60) | <0.0001 |
| Odds ratios are presented per doubling of FGF21 concentration.  Outliers in FGF21 were defined as the highest and lowest 2.5 percentiles.  Model is adjusted for age and sex, BMI, eGFR and urinary albumin excretion, smoking, alcohol intake, hypertension, diabetes, history of cardiovascular disease, HDL cholesterol, LDL cholesterol, triglycerides, plasma albumin concentration.  A history of cardiovascular disease was defined as having a history of coronary artery disease, stroke, heart failure, and/or peripheral artery disease.  Abbreviations: BMI: body mass index; CI: confidence interval; eGFR: estimated glomerular filtration rate; FGF21: fibroblast growth factor 21; HDL: high-density lipoprotein; LDL: low-density lipoprotein; OR: odds ratio. | | | |

| **Table S3.** Sensitivity analyses on the association of FGF21 concentration with low protein intake using additional adjustments. | | |
| --- | --- | --- |
|  | **<0.8 g/day/kg** | |
| **Model** | **OR (95% CI)** | **P-value** |
| Base model | 1.48 (1.38; 1.58) | <0.0001 |
| Base + CERI | 1.40 (1.30; 1.50) | <0.0001 |
| Base + Ketone bodies | 1.48 (1.38; 1.59) | <0.0001 |
| Base + FLI | 1.45 (1.35; 1.55) | <0.0001 |
| Base + HSI | 1.48 (1.38; 1.58) | <0.0001 |
| Base + HOMA-IR | 1.47 (1.37; 1.57) | <0.0001 |
| Base + GlycA and Hs-CRP | 1.46 (1.37; 1.59) | <0.0001 |
| Base + COPD or asthma | 1.46 (1.37; 1.57) | <0.0001 |
| Base + Rheumatic disease | 1.47 (1.37; 1.57) | <0.0001 |
| Base + History of malignancy | 1.47 (1.37; 1.57) | <0.0001 |
| Odds ratios are presented per doubling of FGF21 concentration.  Model is adjusted for age and sex, BMI, eGFR and urinary albumin excretion, smoking, alcohol intake, hypertension, diabetes, history of cardiovascular disease, HDL cholesterol, LDL cholesterol, triglycerides, plasma albumin concentration.  Abbreviations: BMI: body mass index; CERI: creatinine excretion rate index; CI: confidence interval; COPD: chronic obstructive pulmonary disease; eGFR: estimated glomerular filtration rate; FGF21: fibroblast growth factor 21; FLI: fatty liver index; GlycA: glycoprotein acetylation; HOMA-IR: homeostatic model assessment for insulin resistance; HDL: high-density lipoprotein; Hs-CRP: high sensitivity C-reactive protein; HSI: hepatic steatosis index; LDL: low-density lipoprotein; OR: odds ratio | | |

| **Table S4.** Logistic regression analyses of circulating FGF21 with low protein intake using alternative cut-offs, | | | | |
| --- | --- | --- | --- | --- |
|  | **<0.6 g/day/kg** | | **<1.0 g/day/kg** | |
| **Model** | **OR (95% CI)** | **P-value** | **OR (95% CI)** | **P-value** |
| Model 1 | 1.68 (1.51; 1.87) | <0.0001 | 1.48 (1.42; 1.56) | <0.0001 |
| Model 2 | 1.68 (1.51; 1.87) | <0.0001 | 1.43 (1.37; 1.51) | <0.001 |
| Model 3 | 1.67 (1.51; 1.90) | <0.0001 | 1.38 (1.31; 1.45) | <0.001 |
| Model 4 | 1.64 (1.45; 1.87) | <0.0001 | 1.35 (1.29; 1.43) | <0.0001 |
| Events | 229 (4%) |  | 3126 (49%) |  |
| Odds ratios are presented per doubling of FGF21 concentration.  Model 1: Crude.  Model 2: Adjusted for age and sex.  Model 3: As model 2, additionally adjusted for BMI, eGFR and urinary albumin excretion.  Model 4: As model 3, additionally adjusted for smoking, alcohol intake, hypertension, diabetes, history of cardiovascular disease, HDL cholesterol, LDL cholesterol, triglycerides, plasma albumin concentration.  Abbreviations: BMI: body mass index; CI: confidence interval; eGFR: estimated glomerular filtration rate; FGF21: fibroblast growth factor 21; HDL: high-density lipoprotein; LDL: low-density lipoprotein; OR: odds ratio. | | | | |


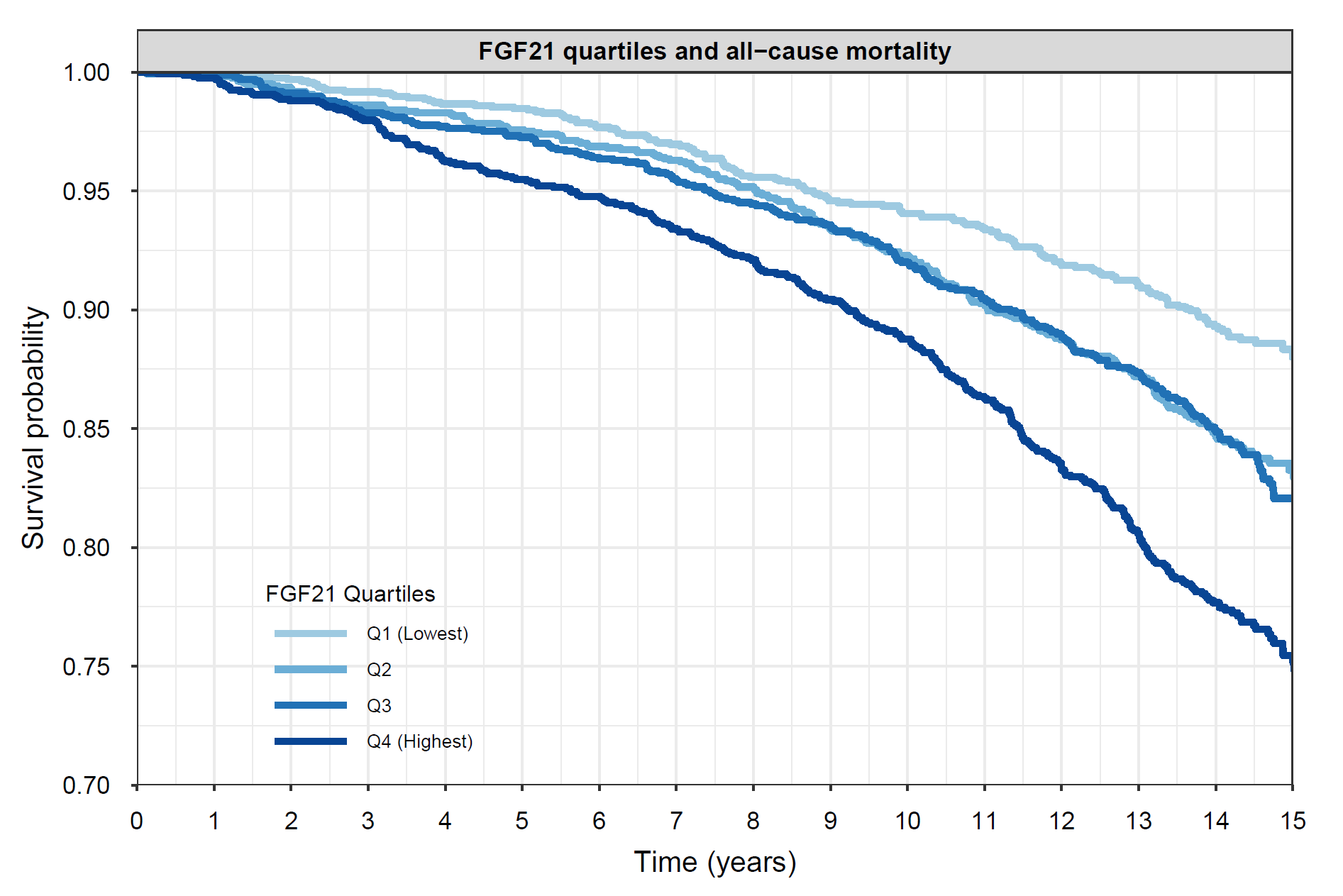


**Figure S4.** Kaplan-Meier survival curves for quartiles of plasma FGF21 levels. The plot displays all-cause mortality stratified by quartiles of circulating FGF21 concentration, with quartile 1 representing the lowest quartile and quartile 4 the highest. The survival probability decreases over time across all quartiles, with a significant difference observed between groups (log-rank test, P<0.001), indicating higher FGF21 levels are associated with a higher risk of mortality.

| **Table S5.** Cox regression analyses with all-cause mortality according to age subgroups. | | | | | | |
| --- | --- | --- | --- | --- | --- | --- |
|  | **FGF21 concentrations** | | **Protein intake** | | **Muscle mass** | |
| **Model** | **HR (95% CI)** | **P-value** | **HR (95% CI)** | **P-value** | **HR (95% CI)** | **P-value** |
| **Subgroup <65 years of age:** | | | | | | |
| Model 1 | 1.29 (1.18; 1.42) | <0.0001 | 0.73 (0.54; 0.97) | 0.03 | 0.89 (0.79; 0.99) | 0.04 |
| Model 2 | 1.17 (1.06; 1.29) | 0.002 | 0.75 (0.56; 1.01) | 0.06 | 0.85 (0.74; 0.96) | 0.012 |
| Model 3 | 1.13 (1.02; 1.25) | 0.015 | 0.69 (0.51; 0.94) | 0.018 | 0.80 (0.69; 0.92) | 0.002 |
| Model 4 | 1.06 (0.95; 1.19) | 0.028 | 0.81 (0.60; 1.10) | 0.17 | 0.85 (0.74; 0.98) | 0.027 |
| **Subgroup ≥ 65 years of age:** | | | | | | |
| Model 1 | 1.27 (1.20; 1.34) | <0.0001 | 0.64 (0.52; 0.78) | <0.0001 | 0.85 (0.78; 0.95) | 0.005 |
| Model 2 | 1.18 (1.09; 1.28) | <0.001 | 0.57 (0.46; 0.70) | <0.0001 | 0.82 (0.74; 0.90) | <0.0001 |
| Model 3 | 1.12 (1.03; 1.21) | 0.008 | 0.55 (0.43; 0.68) | <0.0001 | 0.77 (0.70; 0.85) | <0.0001 |
| Model 4 | 1.10 (1.01; 1.20) | 0.023 | 0.60 (0.48; 0.76) | <0.0001 | 0.82 (0.74; 0.90) | <0.0001 |
| HR is presented per doubling for FGF21 concentration and protein intake and per SD deviation increase for muscle mass.  Model 1: Crude.  Model 2: Adjusted for age, sex and BMI.  Model 3: Further adjusted for eGFR and urinary albumin excretion.  Model 4: Additionally adjusted for smoking, alcohol intake, hypertension, diabetes, history of cardiovascular disease, HDL cholesterol, LDL cholesterol, triglycerides, plasma albumin concentration.  Abbreviations: BMI: body mass index; CI: confidence interval; eGFR: estimated glomerular filtration rate; FGF21: fibroblast growth factor 21; HDL: high-density lipoprotein; HR: hazard ratio; LDL: low-density lipoprotein. | | | | | | |

| **Table S6.** Sensitivity analyses on the association of circulating FGF21 concentration, protein intake and CERI with all-cause mortality in selected subgroups. | | | | | | | |
| --- | --- | --- | --- | --- | --- | --- | --- |
|  |  | **FGF21 concentrations** | | **Protein intake** | | **CERI** | |
| **Model** | **Sample size** | **HR (95% CI)** | **P-value** | **HR (95% CI)** | **P-value** | **HR (95% CI)** | **P-value** |
| Full population | 6395 | 1.09 (1.02; 1.16) | 0.009 | 0.67 (0.56; 0.81) | <0.0001 | 0.83 (0.76; 0.90) | <0.0001 |
| No outliers FGF21 | 6075 | 1.11 (1.02; 1.21) | 0.012 | 0.66 (0.54; 0.80) | <0.0001 | 0.82 (0.75; 0.89) | <0.0001 |
| No outliers protein intake | 6067 | 1.08 (1.01; 1.16) | 0.023 | 0.72 (0.58; 0.90) | 0.003 | 0.86 (0.79; 0.94) | <0.0001 |
| No outliers CERI | 6003 | 1.06 (0.99; 1.14) | 0.12 | 0.74 (0.61; 0.90) | 0.003 | 0.86 (0.79; 0.94) | 0.0005 |
| No participants with diabetes | 6023 | 1.09 (1.01; 1.17) | 0.025 | 0.66 (0.54; 0.80) | <0.0001 | 0.79 (0.72; 0.87) | <0.0001 |
| No BMI <18.5 kg/m^2^ | 6363 | 1.09 (1.02; 1.17) | 0.008 | 0.67 (0.56; 0.81) | <0.0001 | 0.83 (0.77; 0.91) | <0.0001 |
| No BMI >30 kg/m^2^ | 5154 | 1.11 (1.03; 1.19) | 0.006 | 0.66 (0.54; 0.81) | <0.0001 | 0.86 (0.79; 0.94) | 0.002 |
| No eGFR <60 ml/min/1.73m^2^ | 5883 | 1.11 (1.03; 1.20) | 0.004 | 0.69 (0.57; 0.84) | 0.0003 | 0.85 (0.78; 0.93) | 0.0007 |
| No history of cardiovascular disease | 5959 | 1.05 (0.97; 1.14) | 0.21 | 0.70 (0.57; 0.85) | 0.0005 | 0.85 (0.77; 0.93) | 0.0005 |
| Hazard ratios (HRs) are expressed per doubling of circulating FGF21. Analyses are adjusted for age and sex, BMI, eGFR and urinary albumin excretion, smoking, alcohol intake, hypertension, diabetes, history of cardiovascular disease, HDL cholesterol, LDL cholesterol, triglycerides, plasma albumin concentration.  A history of cardiovascular disease was defined as having a history of coronary artery disease, stroke, heart failure, and/or peripheral artery disease.  Abbreviations: BMI: body mass index; CERI: creatinine excretion rate index; FGF21: fibroblast growth factor 21; HR: hazard ratio; CI: confidence interval; eGFR: estimated glomerular filtration rate; HDL: high-density lipoprotein; LDL: low-density lipoprotein | | | | | | | |

| **Table S7.** Sensitivity analyses on the association of circulating FGF21 with all-cause mortality with additional adjustments | | | | | | |
| --- | --- | --- | --- | --- | --- | --- |
|  | **FGF21 concentrations** | | **Protein intake** | | **CERI** | |
| **Model** | **HR (95% CI)** | **P-value** | **HR (95% CI)** | **P-value** | **HR (95% CI)** | **P-value** |
| Base model | 1.09 (1.02; 1.16) | 0.009 | 0.67 (0.56; 0.81) | <0.0001 | 0.83 (0.76; 0.90) | <0.0001 |
| + Ketone bodies | 1.09 (1.02; 1.17) | 0.007 | 0.69 (0.58; 0.83) | <0.0001 | 0.84 (0.77; 0.91) | <0.0001 |
| + FLI | 1.08 (1.01; 1.15) | 0.029 | 0.69 (0.57; 0.83) | <0.0001 | 0.84 (0.77; 0.91) | <0.0001 |
| + HSI | 1.09 (1.02; 1.16) | 0.010 | 0.68 (0.56; 0.81) | <0.0001 | 0.84 (0.77; 0.91) | <0.0001 |
| + HOMA-IR | 1.09 (1.02; 1.16) | 0.013 | 0.67 (0.56; 0.81) | <0.0001 | 0.83 (0.76; 0.90) | <0.0001 |
| + GlycA and Hs-CRP | 1.08 (1.01; 1.15) | 0.031 | 0.68 (0.57; 0.82) | <0.0001 | 0.84 (0.77; 0.91) | <0.0001 |
| + COPD or asthma | 1.08 (1.01; 1.16) | 0.026 | 0.69 (0.57; 0.84) | 0.0001 | 0.85 (0.78; 0.93) | 0.0004 |
| + Rheumatic disease | 1.08 (1.01; 1.16) | 0.024 | 0.68 (0.56; 0.81) | <0.0001 | 0.84 (0.77; 0.92) | 0.0002 |
| + History of malignancy | 1.08 (1.01; 1.16) | 0.021 | 0.68 (0.57; 0.82) | <0.0001 | 0.85 (0.78; 0.93) | 0.0003 |
| HR is presented per doubling for FGF21 and protein intake and per SD deviation increase for muscle mass. Base model is adjusted for age and sex, BMI, eGFR and urinary albumin excretion, smoking, alcohol intake, hypertension, diabetes, history of cardiovascular disease, HDL cholesterol, LDL cholesterol, triglycerides, plasma albumin concentration.  Abbreviations: BMI: body mass index; CERI: creatinine excretion rate index; COPD: chronic obstructive pulmonary disease; CI: confidence interval; eGFR: estimated glomerular filtration rate; FGF21: fibroblast growth factor 21; FLI: fatty liver index; GlycA: glycoprotein acetylation; HDL: high-density lipoprotein; HOMA-IR: homeostatic model assessment for insulin resistance; HR: hazard ratio; HSI: hepatic steatosis index; Hs-CRP: high sensitivity C-reactive protein; LDL: low-density lipoprotein | | | | | | |

| **Table S8.** Sensitivity analyses on the association of circulating FGF21 with all-cause mortality with cause-specific mortality | | | | | | | |
| --- | --- | --- | --- | --- | --- | --- | --- |
|  | | **FGF21 concentrations** | | **Protein intake** | | **CERI** | |
| **Model** | **Events** | **HR (95% CI)** | **P-value** | **HR (95% CI)** | **P-value** | **HR (95% CI)** | **P-value** |
| All-cause mortality | 955 | 1.09 (1.02; 1.16) | 0.009 | 0.67 (0.56; 0.81) | <0.0001 | 0.83 (0.76; 0.90) | <0.0001 |
| Cardiovascular mortality | 242 | 1.01 (0.88; 1.15) | 0.91 | 0.75 (0.51; 1.09) | 0.14 | 0.81 (0.68; 0.95) | 0.012 |
| Non-cardiovascular mortality | 713 | 1.12 (1.04; 1.21) | 0.005 | 0.65 (0.53; 0.81) | <0.0001 | 0.86 (0.78; 0.96) | 0.005 |
| HR is presented per doubling for FGF21 and protein intake and per SD deviation increase for muscle mass. Base model is adjusted for age and sex, BMI, eGFR and urinary albumin excretion, smoking, alcohol intake, hypertension, diabetes, history of cardiovascular disease, HDL cholesterol, LDL cholesterol, triglycerides, plasma albumin concentration.  Abbreviations: BMI: body mass index; CERI: creatinine excretion rate index; FGF21: fibroblast growth factor 21; HR: hazard ratio; CI: confidence interval; eGFR: estimated glomerular filtration rate; HDL: high-density lipoprotein; LDL: low-density lipoprotein | | | | | | | |
